# Supplementary material for: A novel application of neural networks to identify potentially effective combinations of biologic factors for enhancement of bone fusion/repair
Source: PLoS One. 2022 Nov 1;17(11):e0276562. doi: 10.1371/journal.pone.0276562 (PMC9624421; doi:10.1371/journal.pone.0276562)
Supplement: S1 Text — (PDF) [file pone.0276562.s001.pdf]

Text S1: Setting up the training data

Available data were curated from the published literature and organized into a format suitable for machine learning. When possible, metanalyses providing tables listing outcomes for specific therapeutic trials were used for convenience of data collection. When metanalyses did not include appropriate data in table format, specific articles were used to obtain intervention (input) and outcomes (output) data. Literature used specifically to compile the training data can be found at the end of this supplement.[1-12]

After obtaining input and output data, the data were organized into input/desired-output pattern pairs. The inputs were the following medical interventions, with dosage/coding specifications in parentheses: low intensity pulsed ultrasound (total days of post-operative treatment), electrical stimulation (utilization post operatively (1) versus no utilization (0)), rhBMP2 (bone-morphogenic protein; milligrams), rhBMP2 ND (no dose specified, utilized (1) versus not utilized (0)), rhBMP7 (milligrams), autogenous bone graft (utilization intra-operatively (1) versus no utilization (0)), exogenous bone graft (utilization intra-operatively (1) versus no utilization (0)), gelatin-calcium sulfate hydroxyapetite biomaterial (utilization intra-operatively (1) versus no utilization (0)), zoledronic acid (micrograms), osteogenin (micrograms), platelet-derived growth factor (micrograms), platelet-derived growth factor (coated, rat model), bone marrow aspirate concentrate (utilization intra-operatively (1) versus no utilization (0)), human (not human study (0) versus human study (1)), rat (not rat model (0) versus rat model (1)), animal model (not animal model other than rat (0) versus animal model other than rat (eg mouse, horse) (1)), platelet rich plasma (micrograms). Osteoconductive agents (and other bone scaffolds) were included in some studies described in the literature we accessed but we excluded them from the data set except for allograft, as the goal of our study was to characterize biologic factors which could be used in combination within an allograft framework.

The following outputs were outcome measures from clinical and basic science trials, with dosage/coding specifications in parentheses: distraction rate (millimeter per day), bone formation at 3 months (percentage of sample with positive bone formation), bone formation at 6 months (percentage of sample with positive bone formation), mineralized tissue volume divided by total tissue volume, 1-level posterior lumbar fusion percentage (fusion rate percent in sample), Oswestry disability index (ODI) improvement (percentage of sample with an improvement in ODI), fusion rate (percentage of sample with complete bone fusion), fracture healing rate (percentage of sample with complete fracture healing), time to achieve full weight bearing or clinical healing (in days), mean time to radiographic union (in days), Oswestry score (range 0-100), patients satisfied (percentage of patients who are "satisfied" with outcome), need for repeat bone grafting (number of patients), not healed at end of trial (number of patients), need for dynamization (number of patients), radiographic outcome score (assessed by radiologist/orthopedist), histomorphometric/histologic outcome score, implant survival (percent of sample with implant survival), radiopacity (percent of sample with radiopaque bone on radiograph), new bone formation (in mm^2), subsidence (complication occurred (1) versus no complication (0)), graft malposition/loosening (complication occurred (1) versus no complication (0)), infection (complication occurred (1) versus no complication (0)), urogenital adverse events (complication occurred (1) versus no complication (0)), retrograde ejaculation (complication occurred (1) versus no complication (0)), and re-operations (complication occurred (1) versus no complication (0)).

The input and output data were then arranged in a large table using Microsoft Excel.™ A highly abridged portion of the full input-output table is shown in Table 1.

Table 1. Example of the format in which the training data was organized

**input**		**desired output**		
rhBMP2 (mg)	PDGF (ug)	fusion rate (%)	Oswesty score	New bone (mm^2)
14	0	100	18.8	.
5	0	94.5	24.1	.
10	0	98.5	20.9	.
0	20	.	.	0.8
0	60	.	.	0.7
0	200	.	.	1.0

Literature used to compile the training data:

1. Aro, H.T., et al., *Recombinant human bone morphogenetic protein-2: a randomized trial in open tibial fractures treated with reamed nail fixation.* J Bone Joint Surg Am, 2011. **93**(9): p. 801-8.
2. Faundez, A., et al., *Bone morphogenetic protein use in spine surgery-complications and outcomes: a systematic review.* Int Orthop, 2016. **40**(6): p. 1309-19.
3. Gianakos, A., et al., *Bone Marrow Aspirate Concentrate in Animal Long Bone Healing: An Analysis of Basic Science Evidence.* J Orthop Trauma, 2016. **30**(1): p. 1-9.
4. Griffin, X.L., et al., *Electromagnetic field stimulation for treating delayed union or non-union of long bone fractures in adults.* Cochrane Database Syst Rev, 2011(4): p. CD008471.
5. Horstmann, P.F., et al., *(*) Composite Biomaterial as a Carrier for Bone-Active Substances for Metaphyseal Tibial Bone Defect Reconstruction in Rats.* Tissue Eng Part A, 2017. **23**(23-24): p. 1403-1412.
6. Jones, A.L., et al., *Recombinant human BMP-2 and allograft compared with autogenous bone graft for reconstruction of diaphyseal tibial fractures with cortical defects. A randomized, controlled trial.* J Bone Joint Surg Am, 2006. **88**(7): p. 1431-41.
7. Lee, D.H., et al., *Bone marrow aspirate concentrate and platelet-rich plasma enhanced bone healing in distraction osteogenesis of the tibia.* Clin Orthop Relat Res, 2014. **472**(12): p. 3789-97.
8. Marden, L.J., et al., *Platelet-derived growth factor inhibits bone regeneration induced by osteogenin, a bone morphogenetic protein, in rat craniotomy defects.* J Clin Invest, 1993. **92**(6): p. 2897-905.
9. Pocaterra, A., et al., *Effectiveness of platelet-rich plasma as an adjunctive material to bone graft: a systematic review and meta-analysis of randomized controlled clinical trials.* Int J Oral Maxillofac Surg, 2016. **45**(8): p. 1027-34.
10. Rutten, S., et al., *Enhancement of Bone-Healing by Low-Intensity Pulsed Ultrasound: A Systematic Review.* JBJS Rev, 2016. **4**(3).
11. Swiontkowski, M.F., et al., *Recombinant human bone morphogenetic protein-2 in open tibial fractures. A subgroup analysis of data combined from two prospective randomized studies.* J Bone Joint Surg Am, 2006. **88**(6): p. 1258-65.
12. Ye, F., et al., *Comparison of the use of rhBMP-7 versus iliac crest autograft in single-level lumbar fusion: a meta-analysis of randomized controlled trials.* J Bone Miner Metab, 2018. **36**(1): p. 119-127.
