# Supplementary material for: A novel application of neural networks to identify potentially effective combinations of biologic factors for enhancement of bone fusion/repair
Source: PLoS One. 2022 Nov 1;17(11):e0276562. doi: 10.1371/journal.pone.0276562 (PMC9624421; doi:10.1371/journal.pone.0276562)
Supplement: S2 Text — (PDF) [file pone.0276562.s002.pdf]

Text S2: Deep neural networks

The task at hand is to use machine learning to train some form of AI to extract the knowledge contained in an experimental input/desired-output dataset, and then use the AI to generalize on the basis of that knowledge to predict the outcomes of inputs on which it has not been trained. By far the most powerful AIs available for this kind of task are deep neural networks.[1,2] We evaluated several different network types in order to determine which type exhibited the best generalization capability for our dataset.

Deep neural networks are artificial neural networks (ANNS) that have potentially many processing layers. Like real neural networks, ANNS are composed of many interconnected units, which can be thought of as highly simplified neurons. As in the real brain, the units can be organized into layers or circuits. Networks organized into layers are known as feedforward networks, while networks organized into circuits are known as recurrent networks.

The conceptually simplest connectivity pattern for feedforward networks is one in which every unit in a previous layer connects to every unit in the subsequent layer. The conceptually simplest connectivity pattern for recurrent networks is one in which every unit connects to every other unit, forming many circuits. In both cases this pattern is known as complete connectivity. All of the networks we evaluated had complete connectivity.

Each unit in an ANN computes the sum of the activities of all the units that connect to it, after the activity of each sending unit is multiplied by the weight of its connection to the receiving unit. Thus, each unit computes its weighted input sum. The resulting activity of a linear unit will simply be its weighted input sum, unaltered by any further processing. The resulting activity of a nonlinear unit will be the weighted input sum after it has passed through a nonlinear activation function. The most common nonlinear activation function, and the one used here, is the sigmoidal activation function, which maps the real numbers sigmoidally into the range [0, 1]. Because any number of layers of linear units can be mathematically compressed into a single linear layer, all intervening layers in multi-layered neural networks should be nonlinear.

All neural networks have some units that are designed as input units, and others that are designated as output units. The activity levels of the input units are set from outside the network, while the activity levels of the output units are determined from the interactions among all the units in the network. The parameters of a neural network are the weights of the connections between the units. The weights are trained on pairs of input/desired-output patterns using machine learning (ML), so that the network will produce the desired output pattern for each input pattern. The kind of machine learning in which a network is trained to produce a specific output for each input is known as supervised learning.

The simplest artificial neural network has only two layers: input and output; and is trained using the simplest form of supervised learning: the delta rule.[3] The input and output units can be linear. The next simplest artificial neural network has three layers: input, output, and hidden, where the hidden layer intervenes between the input and output layers. The hidden layer is so called because hidden unit activity is neither set (as for input units) nor trained (as for output units). The units in the hidden layer (as in all hidden layers) should be nonlinear.

Deep neural networks have more than one hidden layer and may have as many as ten or more hidden layers. Each additional hidden layer can add further processing power to an artificial neural network, and many layers are needed for certain applications. ANNs with any number of hidden layers can be trained using a generalization of the delta rule known as backpropagation.[3]

Recurrent neural networks have their units arranged in circuits, in which each unit can connect (and here does connect) with every other unit. Because the units can connect in many overlapping circuits, where each unit can both send to and receive from any other unit, recurrent networks process information in time. Recurrent networks are also deep networks, because every time step of processing in a recurrent network is equivalent to a layer in a feedforward network. Recurrent networks are trained using a generalization of backpropagation known as recurrent backpropagation.[4]

Most ML algorithms have parameters that govern their operation. These parameters should be optimized for each network type to ensure the best training over the input/desired-output patterns in the dataset. The three supervised learning algorithms used here (delta-rule, backpropagation, and recurrent backpropagation) train network connection weights on the basis of the difference, or error, between the actual output and the desired output for any input. More specifically, they train the weights using weight-update terms that are derived from the gradient of the error with respect to the weights. The delta-rule, backpropagation, and recurrent backpropagation have two main parameters. The first is the learning rate, which is a positive constant (usually < 1) that is used to scale the weight-update terms. The second is the number of patterns presented to the network before their averaged weight-update terms are used to update the weights. This update-term averaging is known as stochastic gradient descent (SGD), and the number of terms in the SGD average can vary from 1 up to 100 or more.

A special type of feedforward neural network is the autoencoder, which is a neural network that is trained to reproduce the input as its desired output.[5] An autoencoder typically has one nonlinear hidden layer (but it could have several hidden layers) and is trained using backpropagation. The encoding of the input by an autoencoder hidden layer can have various uses, one of which is to serve as the input stage to a feedforward or recurrent neural network. Using an autoencoder encoding of the input, rather than using the raw input itself as the input to a neural network, can improve its performance. More specifically, using an autoencoder encoding as the input to a neural network can improve the ability of the neural network to generalize.

ANNs are valuable as AIs because of their ability to *generalize* the knowledge they extract from a dataset to inputs that are not present in the dataset. Generalization enables a network to predict the outputs to inputs on which it has not been trained. Assessing the ability of a neural network to generalize is straightforward. To do so, the set of input/desired-output patterns is divided into a training set and a testing set (usually in a 75% - 25% split). Then the network is trained only on the input/desired-output patterns in the training set and is tested on the inputs in the testing set. Generalization error is quantified as the total root-mean-squared (RMS) error between the actual outputs and the desired outputs in the testing set. Networks with high generalization capability have low generalization error. Due to the randomness inherent in the ML algorithms we used (delta-rule, backpropagation, and recurrent backpropagation), a proper generalization assessment involves retraining a network of a given type several times and averaging the RMS errors.

References

1. LeCun Y, Bengio Y, Hinton G (2015) Deep Learning. *Nature* **521**: 436.
2. Goodfellow I, Bengio Y, Courville A (2016) *Deep Learning*. MIT press.
3. Rumelhart DE, McClelland JL, PDP Research Group (1986) *Parallel Distributed Processing. Explorations in the Microstructure of Cognition.* MIT Press.
4. Pineda FJ (1989) Recurrent backpropagation and the dynamical approach to adaptive neural computation. *Neural Computation* **2**: 161.
5. Hinton GE, Salakhutdinov RR (2006) Reducing the dimensionality of data with neural networks. Science **313**: 504.
