# Supplementary material for: A novel application of neural networks to identify potentially effective combinations of biologic factors for enhancement of bone fusion/repair
Source: PLoS One. 2022 Nov 1;17(11):e0276562. doi: 10.1371/journal.pone.0276562 (PMC9624421; doi:10.1371/journal.pone.0276562)
Supplement: S3 Text — (PDF) [file pone.0276562.s003.pdf]

Text S3: Finding the right neural network

In principle there are no constraints on the number of neurons in a network, nor on the number of layers in a feedforward network, nor on the number of ways of constraining the connections in feedforward or recurrent networks, nor on any number of other neural network attributes. There is literally an infinite number of neural network types. Still, at least over a limited domain, care should be taken to ensure the suitability of the neural network for any specific application.

We evaluated 8 different neural network types, both with and without an autoencoder as a first stage, for a total of 16 different network configurations. The network types included a simple network having only an input layer and an output layer that was trained using the delta-rule. We called this network Delta. They also included feedforward networks with varying numbers of hidden layers that were trained using backpropagation. We called them BackOne, BackTwo, BackThree, BackFive, BackSeven, and BackTen, which had 1, 2, 3, 5, 7, or 10 hidden layers, respectively. They also included a recurrent network trained using recurrent backpropagation. We called this network Recur. In all networks the input and output units were linear while the hidden units were nonlinear, with their activations confined to the range [0, 1] using the sigmoidal nonlinear activation function.

All algorithms were developed in-house from published descriptions[1-3], written in MATLAB™, and run on Intel Core-i5 based Dell™ workstations. All runtimes pertain to this software and hardware configuration. Some initial evaluations using feedforward networks with 1 or 2 hidden layers showed good generalization capability when they had 100 hidden units per layer and were trained for 50,000 input/desired-output training pattern presentations. During training, patterns are chosen at random with replacement. With 50,000 training cycles (or iterations), each of the 225 input/desired-output patterns in our dataset was presented to the network about 200 times. This number of training cycles was effective in reducing network error without overfitting (Figure S1). 50,000 training iterations required about 15 minutes of computing time for the various ANN types on average. Though there was some dependency on network complexity, MATLAB optimizes matrix computations so computing time depended a lot on data handling, which was basically the same for all the ANN types we considered.

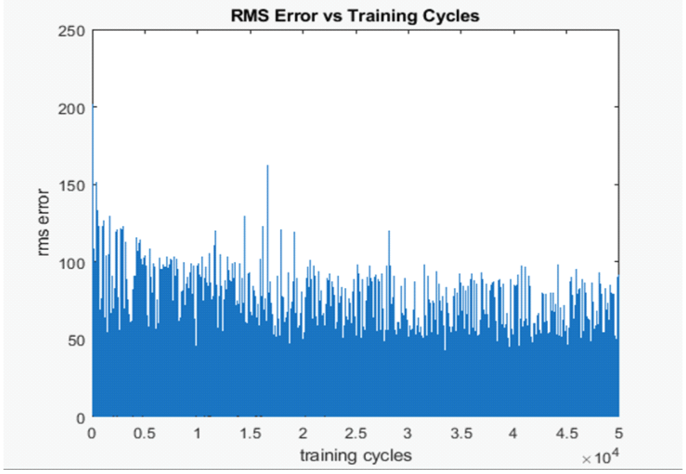

Figure S1. RMS error as a function of training cycles in a representative, feedforward neural network with 1 hidden layer of 100 nonlinear units. RMS error decreases as training proceeds and levels off after about 50,000 training cycles. Error remains relatively high because the dataset contains actual experimental results which differ between different labs, even for the same input values. Halting training after 50,000 training cycles provided adequate error reduction without overfitting the data, allowing good generalization capability.

As shown in Figure S1, RMS error decreases as training proceeds and levels off after about 50,000 training cycles. Since overtraining could compromise network generalization capability, there is no benefit in

training beyond this point. It is clear from Figure S1 that RMS error stays relatively high even after 50,000 training cycles. The reason is that the dataset contains actual experimental results that vary between different labs. For example, the first five input/desired-output entries in the dataset all have exactly the same input values but have five different output values. Clearly, it would be impossible to reduce the error between the desired and actual outputs to zero in this case. Importantly, neural networks are excellent AIs to use in cases where there is ambiguity in the training data, because what they actually learn is to produce an output that is *probably* the best output, given the ambiguity in the dataset.[4]

We next optimized the training parameters for each network type. All hidden layers in feedforward networks had 100 hidden units. Also, there were 100 hidden units in the recurrent network, and it processed the input over 10 time steps. The parameters optimized were the learning rate (a) and the batch size, or the number of weight-update terms that were averaged before they were applied to the weights. The batch size is known as the stochastic gradient descent number (SGDnum) in supervised learning. We found the pair of a and SGDnum that provided the greatest average error reduction over 10 retrainings of each network type. Accomplishing 10 retrainings required about 2.5 hours of computing time on average. The results are shown in Table S1.

**Optimized Parameters of All Network Types without Autoencoder**		
**network type**	**learning rate (a)**	**Stochastic Gradient Descent (SGDnum)**
Delta	0.01	1
BackOne	0.0025	1
BackTwo	0.01	7
BackThree	0.001	1
BackFive	0.001	2
BackSeven	0.00025	2
BackTen	0.0001	2
Recur	0.005	1

Table S1. Optimized learning parameters for all network types. Optimal learning rates (a) and the number of weight-update terms that were averaged before they were applied to update the weights (batch size or SGDnum) could vary considerably between network types.

Having optimized the parameters of our network types, we then assessed the ability of each to generalize. We divided the set of 225 input/desired-output patterns into 175 training patterns and 50 testing patterns. We then retrained each network type 10 times, choosing a different training set and testing set at random each time. The results are shown in Figure S2. They show that a feedforward neural network having one hidden layer generalizes the best.

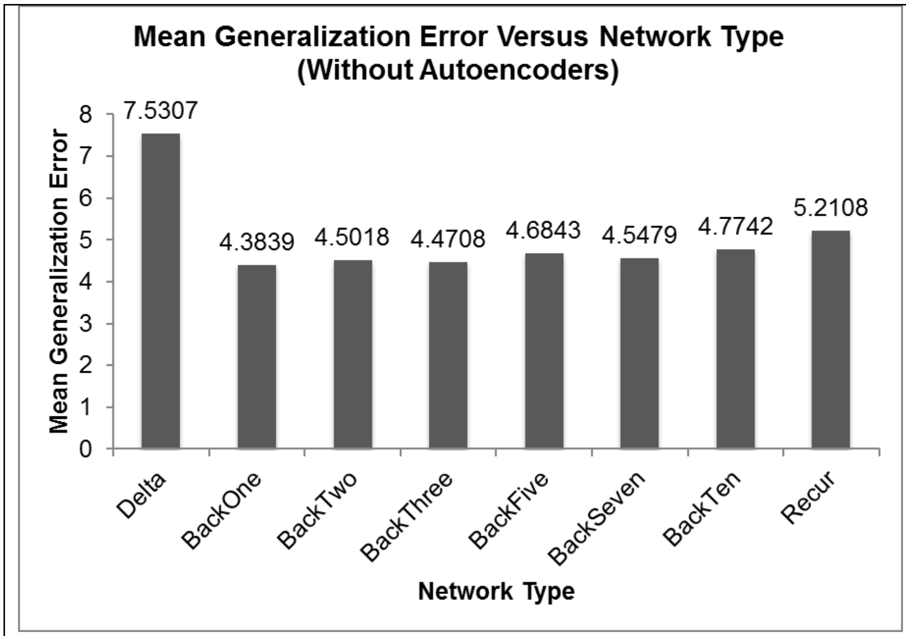

Figure S2. Assessing the ability of neural networks of different types to generalize. Networks of each type were each retrained and retested 10 times, with different input/desired-output patterns chosen each time for the training set and the testing set. The mean generalization error is the average over the 10 retrainings of the RMS error over the testing set. A feedforward neural network having one hidden layer generalizes the best.

We next trained an autoencoder using the input/desired-output values in the dataset. The autoencoder we used had one layer of nonlinear hidden units. We first optimized the training parameters (*a* and SGDnum) for autoencoders having different numbers of hidden units in the single hidden layer (5, 10, 20, 50, or 100). We then assessed the ability of an autoencoder having different numbers of hidden units to generalize over the dataset. Generalization was assessed as described above for the different ANN types. We found that an autoencoder having 50 hidden units showed the best generalization (data not shown).

In many applications, an autoencoder having fewer hidden units than inputs units (a so called undercomplete representation) will have the best generalization capability. Because our dataset has 17 inputs, it was expected that an autoencoder having 5 or 10 hidden units would generalize the best. However, the input in our dataset has three salient characteristics that make it less likely that an undercomplete representation will be optimal. The inputs in our dataset are: sparse, in that most input values are 0 in most input patterns; noisy, in that the desired outputs for the same inputs can vary; and linear, so that different inputs can have different ranges of values (in our case some inputs range from 0 to 1 while others range from 0 to about 15). For these reasons it is not surprising that an overcomplete rather than an undercomplete representation had better generalization properties.[5] We found that an autoencoder having 50 hidden units generalized the best (data not shown).

We next optimized the training parameters for each of the network types with the autoencoded input representation rather than the raw input as the input to the network. The procedures for optimizing the training parameters for the network types with the autoencoder first stage were the same as those described above for the network types without the autoencoder. The results are shown in Table S2.

Optimized Parameters of All Network Types ***With*** Autoencoder		
**network type**	**learning rate (α)**	**Stochastic Gradient Descent (SGDnum)**
Delta	0.05	1
BackOne	0.001	1
BackTwo	0.001	1
BackThree	0.00025	1
BackFive	0.0001	1
BackSeven	0.0005	1
BackTen	0.000025	1
Recur	0.01	1

Table S2. Optimized learning parameters for all network types with an autoencoder first stage. Optimal learning rates (α) varied widely between network types but all types seemed indifferent to the number of weight-update terms that were averaged before they were applied to update the weights (SGDnum). For simplicity, all SGDnum values were set to 1.

Finally, we assessed the ability of each network type with an autoencoder first stage to generalize. The procedure was the same as for the generalizability assessment described above without the autoencoder. The results are shown in Figure S3.

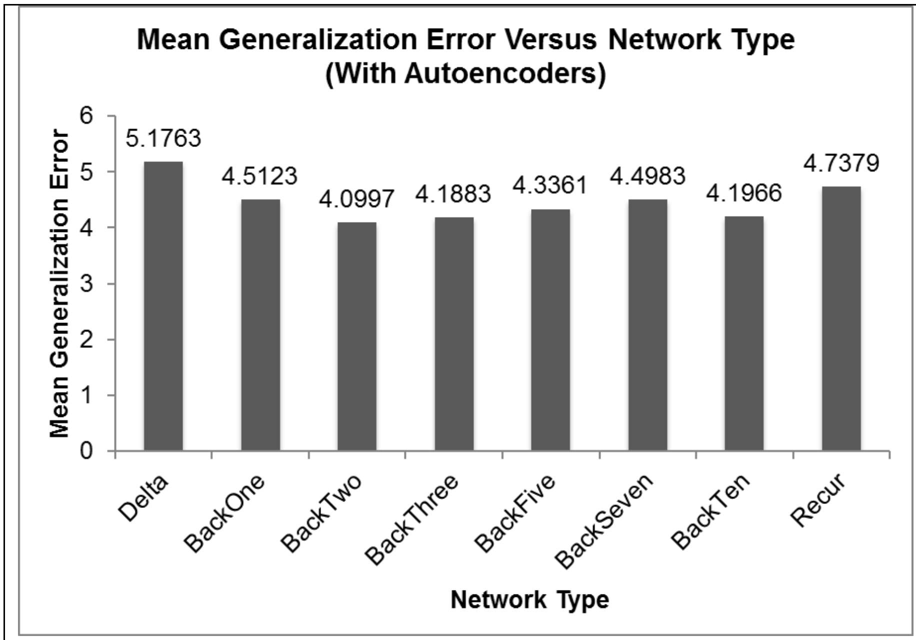

Figure S3. Assessing the ability of neural networks of different types, having an autoencoder as a first stage, to generalize. The generalization assessment was as described in Figure S2. A feedforward neural network with an autoencoder first stage and having two hidden layers generalizes the best. Note that the generalization error for this network is smaller than that for the feedforward network with one hidden layer that generalized the best without an autoencoder (see Figure S2).

With an autoencoder first stage, a feedforward network with two hidden layers showed the best generalization capability. The generalization error for this network is smaller than that for the feedforward network with one hidden layer that showed the best generalization capability for the network without an autoencoder first stage (Figure S2). At 2.5 hours, on average, for 10 retrainings of any ANN type, optimization of the ML parameters for the 16 ANN types required approximately 1 month of computing time. Adding in computing time for preliminary runs and for optimization of autoencoder size and ML parameters, determination of the best ANN for this analysis required about 2 months of computing time. The final result of these assessments is that the network type most suitable for our dataset is a feedforward ANN with two hidden layers, each with 100 hidden units, which also includes an overcomplete autoencoder of 50 hidden units as a first stage. A diagram of this ANN is shown in Figure 1 of the main text.

References

1. Rumelhart DE, McClelland JL, PDP Research Group (1986) *Parallel Distributed Processing. Explorations in the Microstructure of Cognition.* MIT Press.
2. Pineda FJ (1989) Recurrent backpropagation and the dynamical approach to adaptive neural computation. *Neural Computation* **2**: 161.
3. Hinton GE, Salakhutdinov RR (2006) Reducing the dimensionality of data with neural networks. Science **313**: 504.
4. Richard MD, Lippmann RP (1991) Neural network classifiers estimate Bayesian a posteriori probabilities. *Neural Computation* **3**: 461.
5. Vincent P, Larochelle H, Lajoie I, Bengio Y, Manzagol PA (2010) Stacked denoising autoencoders: Learning useful representations in a deep network with a local denoising criterion. *Journal of Machine Learning Research* **11**: 3371.
