# Supplementary material for: A novel application of neural networks to identify potentially effective combinations of biologic factors for enhancement of bone fusion/repair
Source: PLoS One. 2022 Nov 1;17(11):e0276562. doi: 10.1371/journal.pone.0276562 (PMC9624421; doi:10.1371/journal.pone.0276562)
Supplement: S4 Text — (PDF) [file pone.0276562.s004.pdf]

**Text S4: Possible implications of the character of the best network**

The fact that a feedforward neural network with three nonlinear layers (a nonlinear autoencoder layer and two nonlinear hidden layers) generalized best over the dataset could provide insight into the nature of the actual interactions among the biologic factors in the dataset. If all of the biologic factors acted independently, then the simplest network, having only linear input and output layers and trained using the delta rule, would have generalized as well as, if not better than, the more complex network types.

In the simplest network, each output is just a sum of the various inputs, after each input is multiplied by the weight of its connection to the output unit. The simplest network thus implements a linear combination of the inputs, and a linear combination would produce the best generalization in the case in which each input, which is each 1 of the 17 different biologic factors, acts independently. But we found that generalization by the simplest network (Delta) was the worst among the network types we tested, whether with or without an autoencoder first stage.

The fact that generalization was the best in a neural network with three nonlinear layers interposed between the input and output layers indicates that the different orthobiologic factors do not act independently of one another, but instead interact in a complex way. It also suggests that the network was able to learn to represent at least part of the interaction between the different biologic factors, even though it was trained mainly on input/desired-output patterns in which the input included only one active factor. The possibility that the network did learn some of the interactions between the factors lends support to its predictions concerning the efficacies of combinations of the factors. This point is elaborated in the main text.
